# Atrioventricular Block After EVOQUE Transcatheter Tricuspid Valve Replacement

**DOI:** 10.1016/j.shj.2025.100701

**Published:** 2025-07-16

**Authors:** Gautam Rangavajla, Ritik Patel, Felix Nguyen, Brian Vickers, Omar Abdelhai, Brian La Starza, Karl Ilg, Waddah Maskoun, Matthew Ebinger, Marc Lahiri, Arfaat Khan, Josh Greenberg, Georgi Fram, Hussayn Alrayes, Leo Kar Lok Lai, Janet Wyman, Sachin Parikh, Bryan Zweig, John Dawdy, Nathaniel Bowerman, Pedro Engel Gonzalez, William O’Neill, Pedro Villablanca, James Lee, Brian P. O’Neill, Mohamad Raad, Tiberio M. Frisoli

**Affiliations:** a Division of Cardiology, Henry Ford Hospital, Detroit, Michigan, USA; b School of Medicine, Wayne State University, Detroit, Michigan, USA; c Department of Medicine, Henry Ford Hospital, Detroit, Michigan, USA

**Keywords:** AV block, EVOQUE, Heart block, Pacemaker, TTVR

## Abstract

• Based on a real-world study of 106 Evoque transcatheter tricuspid valve replacement (TTVR) recipients, a total of 14 (25%) cases of new atrioventricular (AV) block occurred after Evoque implantation in the 55 patients without prior pacemakers who met study inclusion criteria. This incidence is similar to that reported in the TRISCEND II clinical trial.
• New AV block occurred in a median of 26 hours after Evoque TTVR (interquartile range: 5-60 hours), but there were cases of new AV block that occurred as late as 7 days after the procedure.
• Of the 14 new cases of AV block, 12 were treated successfully with implantation of a leadless pacemaker (Micra) under fluoroscopic guidance without procedural complications or compromising the valve implantation.
• The institutional strategy of 48-hour post-Evoque inpatient monitoring for all patients followed by mobile cardiac outpatient telemetry identified 11 of 14 cases of new AV block as before discharge from the hospital and 1 case as an outpatient before symptom onset.

## Introduction

Transcatheter tricuspid valve replacement (TTVR) offers a minimally invasive approach to treat severe tricuspid regurgitation. The EVOQUE device (Edwards Lifesciences, Irvine, CA) is the first TTVR system approved for commercial use in the United States and improves quality of life.^1^ However, the technology is limited by a 24.7% incidence of postprocedural atrioventricular (AV) block requiring pacemaker implantation.^1^ Understanding this complication is crucial for risk stratification and patient selection. We report our early real-world experiences with AV block after EVOQUE TTVR.

## Methods

This single-center prospective observational study was approved by the local institutional review board and complies with the Declaration of Helsinki. Patients undergoing successful EVOQUE TTVR between device approval on February 1st, 2024, and March 31st, 2025, were included unless they had a pre-existing pacemaker or received a heart transplant within the preceding year. Study data were extracted from the electronic medical record of Henry Ford Hospital in Detroit, Michigan. Key outcomes were the incidence and timing of new high-degree AV block (HDAVB), defined as new complete heart block, Mobitz II block, or atrial fibrillation (AF) with new junctional or ventricular escape rhythms. We also evaluated whether baseline clinical, electrocardiographic, or valvular characteristics were associated with new HDAVB using logistic regression.

The EVOQUE procedure was performed via transfemoral or transjugular access and resulted in placement of a bioprosthetic valve (44, 48, 52, or 56 mm) in the tricuspid annulus. While prior reports^2^ demonstrated the utility of electrophysiologic studies (EPSs) in AV block after transcatheter aortic valve intervention, we did not perform routine EPS during TTVR as EPS (especially measurement of His electrograms) was not anticipated to be feasible with an artificial tricuspid valve and could potentially risk the integrity of valve implantation. Furthermore, we anticipated most patients would have AF,^1^ which would complicate assessment of AV node function.

Following TTVR, patients were monitored per institutional protocol on telemetry for 48 hours in the hospital. Bradycardia on telemetry would prompt an electrocardiogram. If there was clinical concern or electrocardiogram evidence of new HDAVB, the electrophysiology service was consulted for assessment and potentially inpatient pacemaker implantation, with the choice of pacemaker left to the electrophysiologist. Patients with symptomatic bradycardia received either a temporary balloon-tip pacemaker, which required intensive care monitoring at our institution, or a temporary active-fixation lead, which was monitored on telemetry-capable floors. If there was no HDAVB in the first 48 hours, patients were discharged with 30-day mobile cardiac outpatient telemetry (MCOT) to monitor for late-onset HDAVB.

## Results

Of the 106 patients who underwent nonresearch EVOQUE TTVR at our institution, 55 met inclusion criteria. Patients had a mean age of 77 ± 10.8 years, were 75% female, had a mean left ventricular ejection fraction of 56% ± 11%, and 78% had New York Heart Association class III or IV symptoms. A majority (76%) had AF, most with paroxysmal AF (52%) and fewer with persistent AF (24%) or permanent AF (24%).

**Figure 1:**
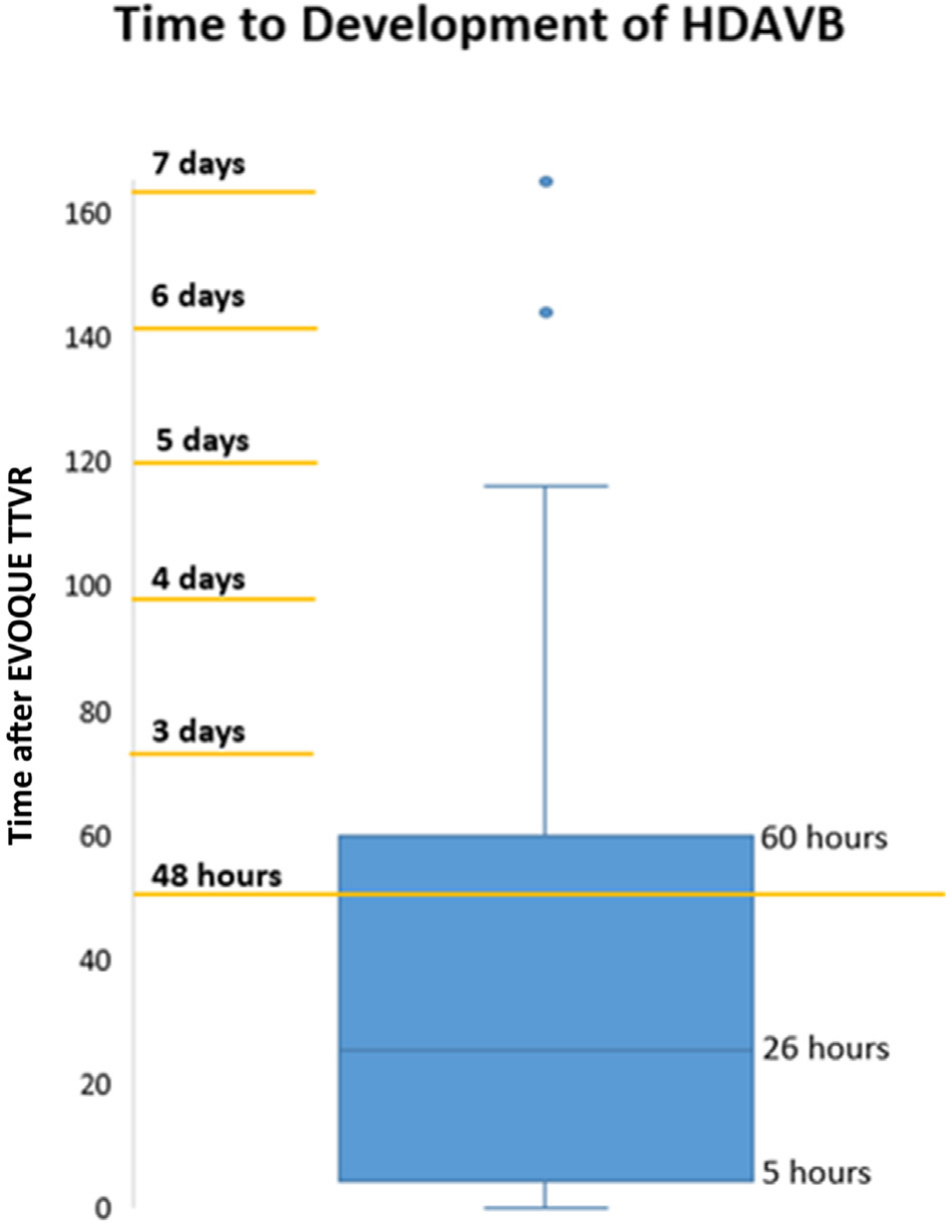
**Patients developed new HDAVB a median 26 hours after EVOQUE TTVR (IQR 5-60 hours)** A total of 3 patients developed HDAVB after the protocolized 48-hour inpatient monitoring period, with the latest onset of HDAVB at 7 days after EVOQUE TTVR. Abbreviations: HDAVB, high-degree atrioventricular block; IQR, interquartile range; TTVR, transcatheter tricuspid valve replacement.

There were 14 (25%) cases of new HDAVB, with onset occurring a median 26 hours after EVOQUE implantation (interquartile range: 5-60 hours), as shown in Figure 1. Late-onset HDAVB after hospital discharge occurred in 3 patients, with 1 identified by MCOT and 2 presenting with syncope or presyncope; all were admitted for pacemaker implantation. There were no significant associations between new HDAVB and baseline left or right bundle branch blocks, tricuspid annulus circumference, EVOQUE valve size or sizing relative to the annulus and basal right ventricle (RV), or other characteristics. Valve oversizing in the annulus (diastole and systole) and RV (diastole and systole) were 7.7% ± 4.5, 11.5% ± 6.0, -5.2% ± 9.5, and 17.0% ± 13.6, respectively.

Of the patients with HDAVB, 1 received left bundle branch area pacing, 1 received middle cardiac vein pacing, and 12 received Micra (Medtronic, Minneapolis, MN) leadless pacing. Leadless pacemaker (LP) implantation is preferred at our institution as it avoids exacerbating tricuspid regurgitation with a trans-tricuspid lead. LP is also appropriate for this patient population, who are largely elderly with preserved left ventricular ejection fraction. Left bundle branch area pacing was implanted in 1 case with partial displacement of the EVOQUE valve due to concern that the Micra delivery sheath could exacerbate the displacement. Middle cardiac vein pacing was used to allow for a conventional pacing system while avoiding a trans-tricuspid lead.^3^ All pacemaker implantations were performed under fluoroscopy with no damage to the EVOQUE valves and no procedural complications.

## Discussion and Conclusion

This study demonstrated a 25% incidence (14 of 55 patients) of new HDAVB after EVOQUE TTVR. While most HDAVB occurred during the 48-hour protocolized monitoring period, 3 cases occurred after hospital discharge. There are as yet no robust associations between new HDAVB and baseline electrocardiographic or valvular characteristics.

This study represents early single-center clinical data and is, to the best of our knowledge, the first and largest report of real-world clinical experiences with HDAVB after EVOQUE TTVR. Our HDAVB incidence is similar to randomized trial data^1^ and while TTVR technology will continue to develop, the high incidence of HDAVB^4^ remains a significant concern. MCOT specifically has utility in monitoring patients after discharge and allowed, in this cohort, for recognition of HDAVB in 1 patient before symptom onset. We propose a post-TTVR HDAVB surveillance strategy of a minimum of 48 hours of inpatient telemetry monitoring followed by 30-day MCOT monitoring in all patients, independent of baseline characteristics. There are limited data to comment on risk stratification using routine EPS after EVOQUE implantation, and the utility of baseline characteristics or post-EVOQUE exercise testing to risk stratify for new HDAVB is also unclear.

With 12 patients receiving successful LP without complications or damage to the EVOQUE valves, we note the efficacy and safety of LP for post-EVOQUE HDAVB. The valve footprint may require a more apical implantation of Micra devices and may complicate implantation of the longer Aveir VR (Abbott, Chicago, IL) LP devices, though the latter is still feasible.^5^

Key limitations include the limited sample size and the single-center nature of the study. With the anticipated growth in TTVR, optimization of surveillance strategies with registry studies is needed to accurately risk-stratify and treat patients with post-TTVR HDAVB.

## Ethics Statement

This research was approved by the local institutional review board and in accordance with ethical guidelines. This study was approved under expedited review without the need for individual-level patient consent, as all data were extracted via review of the electronic medical record without patient contact, and all data extracted were initially collected for clinical care without research-specific testing or interventions.

## Funding

The authors have no funding to report.

## Disclosure Statement

B. P. O’Neill is a consultant to Edwards Lifesciences. T. M. Frisoli is a clinical proctor for Edwards Lifesciences, Abbott, Boston Scientific, and Medtronic. P. Villablanca is a consultant for Edwards Lifesciences and Teleflex. P. E. Gonzalez is a consultant and proctor for Edwards Lifesciences. The other authors had no conflicts to declare.
